# Mapping Information Flows in Urban Traffic Networks: A Transfer Entropy Approach

**DOI:** 10.3390/e28101127

**Published:** 2026-10-14

**Authors:** Vera Pecorino, Giovanni Calabrò, Alessandro Pluchino, Giuseppe Inturri, Salvatore Cavalieri

**Affiliations:** 1 Department of Physics and Astronomy “Ettore Majorana”, University of Catania, Via Santa Sofia 64, 95123 Catania, Italy; vera.pecorino@dfa.unict.it (V.P.); alessandro.pluchino@ct.infn.it (A.P.); 2 Department of Electrical, Electronic and Computer Engineering, University of Catania, Via Santa Sofia 64, 95123 Catania, Italy; giuseppe.inturri@unict.it (G.I.); salvatore.cavalieri@unict.it (S.C.); 3 INFN Sezione di Catania, Via Santa Sofia 64, 95123 Catania, Italy

**Keywords:** complex networks, traffic monitoring, floating car data, information theory

## Abstract

This study applies Transfer Entropy (TE) to floating car data from the urban road network of Catania (Italy) to investigate directional dependencies in traffic flows and identify relevant patterns of information transfer. Unlike traditional correlation-based approaches, TE captures asymmetric directed information dependencies between road segments, enabling the identification of predictive relationships among congestion states across the network. We constructed TE-based influence networks using traffic speed data collected over 15 min intervals across 86 road segments over six weeks. The analysis examined information transfer using history lengths of 1, 2, and 3 observations, corresponding to 15, 30, and 45 min of past information, revealing distinct patterns between time windows. Key findings include the identification of recurring information-transfer relationships across multiple history lengths, and road segments with comparatively high outgoing or incoming information strength. Notably, the morning and evening time windows differ in network density, total strength and highest-ranked nodes, although some of these patterns depend on the discretisation and history length adopted, highlighting the dynamic nature of urban traffic systems. This approach shifts focus from traditional traffic prediction to network prioritisation, allowing the identification of candidate locations for enhanced traffic monitoring with minimal data requirements. The methodology provides practical insights for optimising sensor placement and informing further traffic-management analysis based on predictive influence patterns rather than congestion levels alone.

## 1. Introduction

Urban traffic systems play a crucial role in modern cities, directly impacting economic productivity, environmental quality, and citizen well-being. These systems are increasingly complex, shaped by fluctuating demand, spatial constraints, and the need for real-time operational decisions. Managing congestion effectively requires more than identifying where congestion occurs; it also requires characterising the directional temporal dependencies that connect traffic states across different parts of the road network.

Traditional traffic models, including traffic assignment and macroscopic simulation approaches, often rely on assumptions about flow equilibrium or static capacities, which can limit their ability to capture dynamic interactions between different locations. While data-driven approaches have gained popularity thanks to the widespread availability of real-time data from fixed sensors and GPS-enabled vehicles, most of these methods still analyse road segments independently or rely on symmetric statistical measures such as correlation [1,2]. Crucially, correlation describes co-variation but not directional influence—a fundamental limitation for understanding how traffic conditions spread through networks.

This inability to capture directionality represents a critical gap in the characterisation of traffic networks. Correlation and similar symmetric dependency measures cannot determine whether the past state of one location provides predictive information about the future state of another. As extensively discussed in the literature on dynamical systems and complex networks [3], distinguishing directional temporal dependencies from simple co-fluctuations is therefore important for network-level traffic analysis. Without such information, predictive models may capture statistical co-variation while overlooking asymmetric temporal relationships between road segments, thereby limiting their usefulness for network monitoring and prediction.

Recognising these limitations, several studies have explored information-theoretic approaches to traffic analysis. Granger causality, for instance, has been applied to traffic time series to infer directional dependencies [4], but its linear assumptions often prove inadequate for highly non-linear traffic systems. Information entropy-based methods offer a more promising alternative, providing model-free approaches to assess uncertainty and information transfer in traffic flows [5]. Among these methods, Transfer Entropy stands out for its ability to detect asymmetric temporal dependencies without requiring strong assumptions about the underlying dynamics [6]. However, despite its theoretical potential, there are only a limited number of applications of TE to urban traffic systems at the network level. Our objective is to identify directional information-transfer relationships across the urban network and to determine which locations consistently act as information sources or sinks. Building upon network-based approaches previously used for precipitation time series [7], we construct directed, weighted networks across multiple history lengths and temporal windows. This approach reveals dynamic information-dependence patterns that may inform strategic decisions concerning traffic monitoring, sensor deployment, and short-term prediction. Fundamentally, this perspective shifts the focus from identifying where congestion occurs to characterising how predictive information dependencies are organised across the network.

The remainder of this paper is organised as follows. Section 2 reviews the state of the art in traffic modelling, emphasising the transition from traditional and data-driven approaches to information-theoretic frameworks. Section 3 presents the conceptual contribution of this work, reframing traffic analysis as a problem of directional information transfer. Section 4 describes the proposed methodology, including the formulation of Transfer Entropy, the definition of the Traffic Speed Index, and the construction of directed information-transfer graphs. Section 5 illustrates the case study of Catania, detailing the analysis setup, results, and discussion of empirical findings and outlines future research directions. Finally, Section 6 summarises the main outcomes.

## 2. State of the Art

Traditional approaches to traffic modelling can be broadly classified into model-based and data-driven frameworks. Classical assignment and simulation models were developed to reproduce the interaction between travel demand and network capacity under assumed equilibrium conditions [8]. Although such models provide valuable insights for long-term planning, their reliance on behavioural and static assumptions—such as rational route choice and fixed trip rates—limits their ability to capture the rapid and spatially heterogeneous dynamics that characterise real-world urban congestion [9,10].

The availability of large-scale mobility data has progressively shifted research toward data-driven prediction. Statistical filtering techniques [11], machine learning frameworks [12], and deep learning architectures [13] have demonstrated impressive predictive power when trained on extensive historical datasets [14,15]. However, these models typically optimise forecasting accuracy without explicitly representing the mechanisms that generate congestion. By relying on correlation or symmetric similarity measures, they tend to overlook the directional nature of interactions between road segments, where disturbances in one location can trigger delayed effects elsewhere [1].

To move beyond purely predictive paradigms, recent studies have emphasised the importance of uncovering the structural dependencies underlying traffic dynamics. This emerging research direction integrates concepts from network science and information theory [16,17], seeking to represent traffic as a complex adaptive system in which information about congestion propagates through directed pathways rather than static flows.

Within this context, methods for inferring directional dependencies have gained increasing attention. Classical frameworks such as Granger causality [18] make it possible to detect delayed dependencies between time series but are inherently limited by their linearity assumption. Non-linear alternatives derived from information theory provide a model-free framework to capture more general forms of dependency. Among them, Transfer Entropy (TE), introduced by Schreiber [19], has become a key tool to quantify directional information flow between stochastic processes. TE extends Shannon entropy by measuring how much the past of one process improves the predictability of another, thus revealing asymmetric and directed relationships. Unlike correlation or linear causality tests, TE requires no assumption of linearity, stationarity, or Gaussianity, making it particularly suited to traffic systems characterised by non-linearity and feedback mechanisms. Its interpretation as a measure of effective connectivity rather than physical causation underscores its relevance for analysing complex urban dynamics [20].

Building on these methodological advances, several works have explored the use of TE for traffic analysis. Yang et al. [6] introduced a dynamic spatio-temporal TE framework to reveal evolving causal relationships among road segments, introducing a separate prediction-horizon parameter to keep embedding length distinct from delay, applied to a single expressway network. Oh et al. [21] treated the source–target lag itself as the quantity of interest, estimating it together with a bootstrap-based procedure for uncertainty quantification, to reconstruct accident-triggered congestion-propagation delays along specific paths of adjacent roads. More recently, Dong et al. [22] proposed a large-scale causal-discovery approach that combines a multivariate structural model with an information-criterion-guided selection step to identify influential nodes and mediators within urban networks, operating on traffic subprocesses obtained through a spatial dimensionality-reduction step rather than on individual road segments. Liu et al. [5] likewise adopt an information-theoretic perspective, but characterise the relationship between speed, density and uncertainty at the level of individual segments, without addressing directionality or network-wide propagation. These studies collectively demonstrate the potential of TE and related information-theoretic measures to uncover directional dependencies that reflect how congestion information spreads through space and time.

At the same time, advances in data-driven network modelling—such as Graph Multi-Attention Networks [23] and LSTM-based spatio-temporal models [13]—have begun to incorporate similar principles of adaptive connectivity, implicitly aligning with the causal interpretation of information flow. Together, these developments mark a conceptual shift in traffic research: from predicting states to explaining interactions. Within this emerging paradigm, Transfer Entropy provides a quantitative bridge between statistical learning and network-level understanding, offering a means to move beyond prediction towards the characterisation of directed information dependencies and the identification of candidate locations for traffic monitoring in complex urban traffic systems.

## 3. Contribution

Traditional traffic assignment models aim to capture aggregate network patterns based on assumed knowledge of road segment capacities, cost functions, and origin–destination flows. Instead, data-driven traffic prediction models have typically treated road segments as isolated elements or relied on correlation measures.

This study advances traffic network analysis by reframing it as a problem of directional information dependence rather than aggregate flow measurement. Rather than proposing a new entropy formulation, our contribution lies in developing a systematic network-level framework that transforms sparse floating car data into information-transfer maps. These maps can identify road segments that exhibit comparatively source-like or target-like informational roles, independently of their physical adjacency, and can therefore highlight candidate locations for enhanced monitoring and further traffic-management analysis. Rather than asking whether two segments are congested at the same time, we ask whether the past state of one segment provides predictive information about the future state of another. This shift from traffic prediction to network-level information analysis provides a resource-efficient framework for prioritising monitoring locations on the basis of directional temporal dependencies rather than congestion levels alone.

Relative to prior TE-based studies, our framework differs along four methodological dimensions. First, while Yang et al. [6] and Oh et al. [21] both set k=l=1, we explicitly assess the sensitivity of the inferred networks to the bin count and target-history length, distinguishing patterns that remain stable across the explored settings from those that depend on these parameters. Second, the TSI normalisation enables comparability across heterogeneous urban road segments with different speed limits and geometries, a requirement that does not arise in the single-facility motorway setting of Yang et al. [6], the accident-triggered path analysis of Oh et al. [21], or the segment-level entropy analysis of Liu et al. [5]. Third, unlike Dong et al. [22], whose causal-discovery step operates on spatially aggregated traffic subprocesses, our framework retains full segment-level resolution across the whole network, prioritising direct spatial interpretability and sensor-placement guidance over the multivariate disentanglement of hidden common factors, a trade-off discussed explicitly in Section 5.4. Finally, by contrasting morning and evening time windows on the same infrastructure, it also captures source/target role variations that none of these single-period or single-event studies is designed to reveal.

As regards the traffic analysis, while most approaches rely on fixed sensor infrastructures with inherent spatial limitations and high deployment costs, we leverage ubiquitous GPS speed data to achieve network-wide influence mapping, without requiring costly infrastructure or direct vehicle flow measurements. High-resolution sensors (e.g., licence plate recognition, UAV monitoring) can directly capture vehicle flows, but they are either spatially limited or prohibitively expensive. Our TE-based framework extracts temporal dependencies that reveal influence patterns even from sparse, passively collected FCD. This framework can thus guide strategic sensor placement by identifying which segments warrant enhanced monitoring based on their informational role rather than congestion levels alone.

We introduce a Traffic Speed Index (TSI) indicator that aggregates mean speeds from FCD samples and serves as input for TE computations. By normalising each segment against its own free-flow speed (derived from night-time observations, not legal limits), the TSI enables direct comparison of congestion intensity across roads with vastly different operational characteristics. This indicator captures traffic state variations across network segments without requiring assumptions about underlying flow relationships or equilibrium conditions. The TSI provides an intuitive measure of congestion intensity: values approaching zero indicate traffic approaching road capacity (high saturation), while values near or above one represent free-flow conditions.

We further reformulate traffic analysis from a problem focused exclusively on prediction accuracy to one that also characterises the informational role of individual road segments within the network. Rather than identifying “control points” or assuming that particular segments physically drive network dynamics, the proposed framework ranks segments according to their incoming and outgoing TE-based information strength. This allows the identification of locations that behave as information sources, information sinks, or exhibit dual roles that vary between time windows. Such rankings are interpreted as indicators of predictive informational relevance and as a basis for prioritising monitoring, rather than as evidence of direct physical causation or intervention effects.

By integrating tools from information theory, complex networks, and urban traffic engineering, our method offers an interdisciplinary contribution that advances current approaches to sustainable and adaptive traffic management. The proposed workflow is generalisable to any large-scale network with FCD-type data and does not depend on pre-existing road topology information. In this sense, the work contributes a scalable, topology-agnostic framework for exploring spatio-temporal information flows in urban systems. In doing so, it opens new perspectives on how minimal data sources can yield high-impact insights for planning and decision-making.

## 4. Methodology

### 4.1. Transfer Entropy

Transfer Entropy (TE) is an information-theoretic measure introduced by Schreiber [19] to quantify the directional information flow between two stochastic processes. Unlike mutual information, which captures undirected statistical dependencies, TE explicitly accounts for the temporal structure of the data, thus enabling the detection of directed information dependencies in a dynamical sense.

Consider two discrete stochastic processes *X* and *Y* representing time-series data. Transfer Entropy $TE_{X\to Y}$ from *X* (source process) to *Y* (target process) quantifies the reduction in uncertainty about the future state of *Y* given knowledge of past states of *X*, beyond the information already contained in the history of *Y* itself. This can be expressed as follows:

(1) $$TE_{X\to Y}=\sum_{t\in T}p\left(y_{t+1},y_{t}^{\left(k\right)},x_{t}^{\left(l\right)}\right)\log\frac{p(y_{t+1}|y_{t}^{\left(k\right)},x_{t}^{\left(l\right)})}{p(y_{t+1}|y_{t}^{\left(k\right)})}$$

where $x_{t}$ and $y_{t}$ represent the state value of *X* and *Y* at the time *t*, respectively; $y_{t}^{\left(k\right)}=\left[y_{t},y_{t-1},\ldots ,y_{t-k+1}\right]$ and $x_{t}^{\left(l\right)}=\left[x_{t},x_{t-1},\ldots ,x_{t-l+1}\right]$ denote the past *k* and *l* states of the target and source processes, respectively; and $p(y_{t+1}|y_{t}^{\left(k\right)},x_{t}^{\left(l\right)})$ and $p(y_{t+1}|y_{t}^{\left(k\right)})$ measure the conditional probabilities. The summation is taken over the joint probability distribution $p(y_{t+1},y_{t}^{\left(k\right)},x_{t}^{\left(l\right)})$ of these state values.

### 4.2. Traffic Speed Index for Transfer Entropy Analysis

To apply Transfer Entropy to traffic flow analysis, we require a standardised measure that captures traffic state variations across different road segments and time windows. To characterise congestion levels in a dimensionless form suitable for TE computation, we define the Traffic Speed Index (TSI) as follows:

(2) $$TSI_{t}^{\left(i\right)}=\min\left(1,v_{t}^{\left(i\right)}/v_{0}^{\left(i\right)}\right)$$

where $v_{t}^{\left(i\right)}$ is the average speed for segment *i* during time interval *t*, and $v_{0}^{\left(i\right)}$ is the free-flow speed for segment *i*. The free-flow speed $v_{0}^{\left(i\right)}$ is calculated as the mean of average speeds recorded during night-time intervals (00:00–06:00) for segment *i*, with outliers removed using Tukey’s interquartile range (IQR) method, ensuring a robust estimate of typical free-flow conditions. Specifically, speed observations below $Q_{1}-1.5\times IQR$ or above $Q_{3}+1.5\times IQR$ are excluded, where $Q_{1}$ and $Q_{3}$ are the first and third quartiles, respectively, and $IQR=Q_{3}-Q_{1}$.

The resulting TSI ranges from 0 (complete congestion) to 1 (free-flow conditions), providing a standardised measure of traffic state that accounts for segment-specific characteristics. By normalising each segment against its own empirically observed free-flow speed rather than legal limits, the TSI captures real operational conditions whilst enabling cross-segment comparability: a TSI value of 0.5 represents equivalent relative congestion (in terms of travel delays) regardless of road type. Importantly, road segments were selected to ensure operational homogeneity (urban roads within the city centre with speed limits ≤50 km/h, serving primary network functions), excluding motorways and segments with significant traffic control discontinuities. This segment-specific normalisation ensures that TE calculations are comparable across road segments with different speed limits and geometric characteristics, while the dimensionless nature of TSI facilitates the discretisation process required for information-theoretic analysis across heterogeneous road networks.

### 4.3. Data Processing and Information-Transfer Graph Construction

TE is employed to characterise directional predictive dependencies among traffic time series sampled every 15 min. We evaluate target-history lengths k∈{1,2,3}, while fixing the source-history length to l=1 in Equation (1), consistently with the discrete Transfer Entropy implementation used in the analysis. Thus, *k* controls the amount of target history conditioned upon, whereas the source contributes its most recent past state. In all cases, the predicted state is one 15 min step ahead. This analysis transforms the road network into a directed information-transfer graph, where each road segment represents a node and TE values exceeding the empirical surrogate threshold form weighted edges indicating the strength of information transfer between locations.

Here, TE-based information transfer refers to temporal information transfer, i.e., the statistical improvement in predicting one segment’s future state from another segment’s past, rather than physical vehicle flows or direct physical causation. This interpretation aligns with the notion of effective connectivity in dynamical systems [20] and its application to spatio-temporal traffic dependencies [6], recently extended to large-scale causal-network inference [22].

To ensure data quality and consistency, a pre-processing routine was implemented. Missing observations, represented by zero-valued entries in the original dataset, were replaced using the mean of the closest preceding and succeeding non-zero observations. When only one neighbouring observation was available, its value was used instead. Denoting with *N* the number of analysed nodes and with *T* the number of time steps, the TSI time series are stored, for each analysis time window, in a matrix $X\in R^{N\times T}$.

Since TE is estimated from discrete states, the continuous TSI values were converted into discrete categories through a binning procedure. The discretisation process involves two parameters that affect TE estimation: the number of bins *b* and the history length *k*. The former determines the resolution of the discretised traffic states: fewer bins provide a coarser representation, whereas a larger number of bins increases state-space resolution but also requires more observations for reliable probability estimation. The latter defines the history length, or embedding dimension, i.e., the number of past time steps included in the state history used to compute TE. Therefore, increasing *k* increases the amount of past information included in the TE calculation and should not be interpreted as increasing the physical propagation delay between source and target segments.

A quantile-based global binning strategy was applied to the entire set of TSI values across all road segments. This approach ensures comparability between locations and avoids biases introduced by uneven sampling or segment-specific scaling, following procedures similar to those adopted in network-reconstruction studies [24]. Alternative adaptive-binning methods may maximise mutual information locally but hinder cross-node comparability when applied to multivariate data [25]. For this reason, the global-quantile approach was adopted as a conservative and reproducible choice for network-level analysis.

We assessed discretisation sensitivity over b∈{4,5,6,7} and target-history lengths k∈{1,2,3}. Raw mean TE was not used as an optimisation criterion, because both observed and surrogate TE increased with the dimensionality of the discrete state space. Instead, b=4 was adopted as the primary, least-sparse specification, and b=5,6,7 were treated as sensitivity settings. The primary network analysis uses k=1. Results for k=2 and k=3 are retained as exploratory sensitivity analyses because the number of possible joint states increases rapidly while the effective sample size falls from 798 to 714 observations per directed pair, as shown in Table 1.

TE estimation is inherently sensitive to sampling limitations and discretisation bias. When the number of observed samples approaches or falls below the number of possible discrete states, entropy estimates can become systematically biased. Analytical corrections [26,27] and Bayesian formulations [28] have been proposed to address this issue, substantially improving the reliability of information-theoretic inference in short or unevenly sampled time series.

Across discretisations, the ranking of the strongest source nodes was more stable than the exact network topology. For k=1, comparing b=4 with b=7 yielded source-strength Spearman correlations of 0.924 in the morning and 0.800 in the evening, whereas edge-set Jaccard indices were 0.436 and 0.214, respectively. Accordingly, conclusions are restricted to patterns that remain stable across the sensitivity analysis.

For a given history length *k*, TE can only be computed for time steps for which the required past states are available. This defines the valid time-index set

$$T_{\mathrm{valid}}^{\left(k\right)}\subseteq \left\{k,\ldots ,T-1\right\}.$$

The validity of TE-derived dependencies was assessed through surrogate-data analysis. For each history length and time window, surrogate TE values were obtained by randomly permuting the source time series while leaving the target time series unchanged. This procedure preserves the marginal distribution of the source while destroying its temporal ordering and its temporal dependence with the target. The resulting surrogate TE values were pooled into a global null distribution, and its 98th percentile was used as an empirical network-level filtering threshold for the corresponding history length and time window. Because random permutation disrupts the temporal ordering of the source series, temporal autocorrelation is not preserved in the surrogate data.

For each history length *k*, the corresponding empirical threshold is therefore defined as

(3) $$\theta^{\left(k\right)}=Q_{0.98}\left(D^{\left(k\right)}\right),$$

where $D^{\left(k\right)}$ denotes the pooled surrogate TE distribution and $Q_{0.98}$ its 98th percentile.

An observed directed connection from segment *j* to segment *i* is retained only when

(4) $${\mathrm{TE}}_{j\to i}^{\left(k\right)}>\theta^{\left(k\right)}.$$

Accordingly, the weighted adjacency matrix for history length *k* is defined as

(5) $$A_{ij}^{\left(k\right)}=\left\{\begin{array}{cc} {\mathrm{TE}}_{j\to i}^{\left(k\right)},\; & {\mathrm{TE}}_{j\to i}^{\left(k\right)}>\theta^{\left(k\right)},\\ 0,\; & \mathrm{otherwise}. \end{array}\right.$$

The adopted procedure relies on a global empirical surrogate threshold rather than on edge-specific hypothesis tests and associated *p*-values. Consequently, no Benjamini–Hochberg false-discovery-rate correction was applied. The 98th-percentile criterion is therefore interpreted as an empirical network-level filtering threshold rather than as an edge-wise p<0.02 significance level.

Sampling uncertainty in the principal network-level summaries was assessed for the primary b=4, k=1, l=1 specification using a leave-one-day-out block jackknife. Each of the 42 calendar days was omitted in turn, while all 15 min observations and all 86 road segments belonging to the remaining days were retained together. For every jackknife replicate, the global quantile boundaries, observed TE matrix, pooled surrogate distribution, 98th-percentile filtering threshold, and network summaries were re-estimated. Two-sided 95% confidence intervals were calculated from the jackknife standard errors using the *t* critical value with 41 degrees of freedom. These intervals quantify sensitivity to day-to-day sampling variation under the implemented network-filtering procedure and are not edge-wise significance intervals.

Finally, consistency across history lengths was assessed by identifying information-transfer relationships that are retained for all k∈{1,2,3}. Here, consistency refers to the recurrence of the same directed information-transfer relationship across different amounts of past information and should not be interpreted as persistence across different physical propagation delays.

The complete procedure, outlined in Algorithm 1, was applied independently to each analysis time window. The resulting directed and weighted networks encode effective rather than physical connectivity: each retained edge represents a directed temporal dependency in which the past state of one segment provides predictive information about the future state of another, rather than evidence of direct physical causation.
**Algorithm 1** Transfer Entropy and Adjacency Matrix Construction  1:**Input:** $X\in R^{N\times T}$ time-series matrix, number of bins *b*, maximum history length *K*, number of nodes *N*, number of time steps *T*  2:Discretise X into *b* states using global quantile boundaries  3:Initialise the set of weighted adjacency matrices A←∅  4:**for** k∈{1,2,…,K} **do**  5:       Determine the valid time-index set $T_{\mathrm{valid}}^{\left(k\right)}$  6:       Initialise the surrogate null distribution $D^{\left(k\right)}\leftarrow \emptyset$  7:       **for** i=1 to *N* **do**  8:              **for** j=1 to *N*, j≠i **do**  9:                    Randomly permute the source time series:$${\tilde{X}}_{j}\leftarrow \mathrm{permute}\left(X_{j}\right)$$10:                    Compute surrogate Transfer Entropy$${\tilde{\mathrm{TE}}}_{j\to i}^{\left(k\right)}=\mathrm{TE}\left({\tilde{X}}_{j},X_{i};k\right)\;\mathrm{over}T_{\mathrm{valid}}^{\left(k\right)}$$11:                    Add ${\tilde{\mathrm{TE}}}_{j\to i}^{\left(k\right)}$ to $D^{\left(k\right)}$12:              **end for**13:       **end for**14:       Set the empirical filtering threshold$$\theta^{\left(k\right)}\leftarrow Q_{0.98}\left(D^{\left(k\right)}\right)$$15:       Initialise $A^{\left(k\right)}\leftarrow 0_{N\times N}$16:       **for** i=1 to *N* **do**17:              **for** j=1 to *N*, j≠i **do**18:                    Compute the observed Transfer Entropy$${\mathrm{TE}}_{j\to i}^{\left(k\right)}=\mathrm{TE}\left(X_{j},X_{i};k\right)\;\mathrm{over}T_{\mathrm{valid}}^{\left(k\right)}$$19:                    **if** ${\mathrm{TE}}_{j\to i}^{\left(k\right)}>\theta^{\left(k\right)}$ **then**20:                           $A^{\left(k\right)}\left[i,j\right]\leftarrow {\mathrm{TE}}_{j\to i}^{\left(k\right)}$21:                    **end if**22:              **end for**23:       **end for**24:       Add $A^{\left(k\right)}$ to A25:**end for**26:**Output:**$$A=\left\{A^{\left(1\right)},A^{\left(2\right)},\ldots ,A^{\left(K\right)}\right\}$$

## 5. Case Study

The proposed methodology is applied to the case study of Catania (Italy), a major metropolitan area characterised by significant traffic congestion, particularly during peak hours. The analysis focuses on the city centre’s urban road network, where all streets have speed limits of 50 km/h or below. With its frequent intersections and complex traffic interactions, this urban environment provides a suitable setting for examining directional information dependencies across spatially distributed road segments. Characterising such dependencies may support traffic monitoring and further traffic-management analysis in similar urban contexts.

### 5.1. Analysis Setup

**Figure 1:**
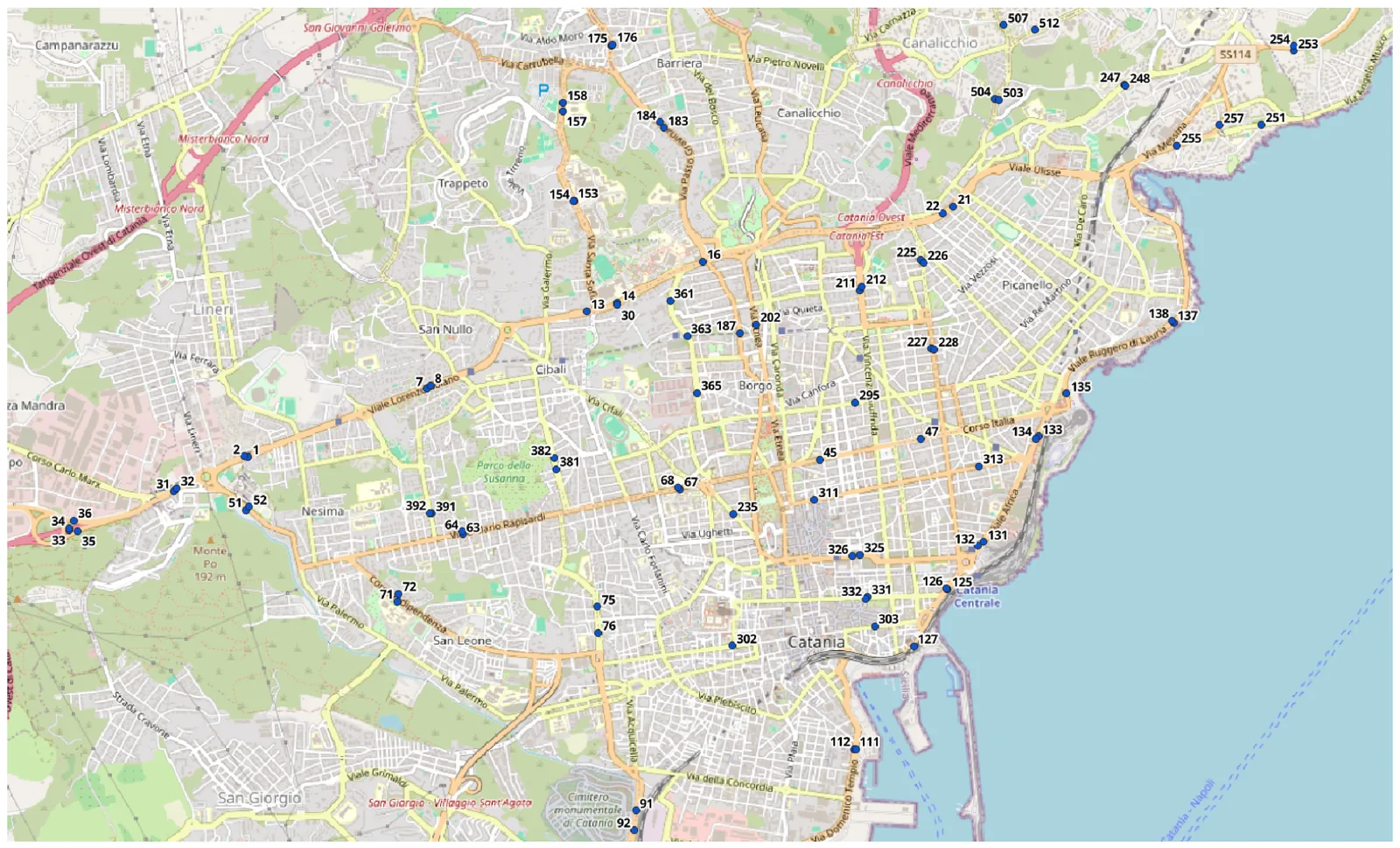
Spatial distribution of the network nodes (with their numerical IDs) analysed in this study. Overlapping markers indicate road segments with opposite traffic directions on the same physical infrastructure.

The traffic time series analysed in our study consist of 15 min interval measurements across 86 road segments—which are shown in Figure 1. The data cover the period from 18 September 2023 to 29 October 2023 and represent GPS-based speed measurements provided by TomTom’s FCD platform [29]. A complete listing of the 86 road segments, including street name, functional road class, geometric attributes, coordinates, and data-availability indicators, is provided in Appendix A (Table A1).

The TomTom dataset is derived from a sample of GPS-enabled vehicles representing a fraction of the total traffic flow. The 42-day study period included both weekdays and weekends. No distinction was made between weekday and weekend observations: all days were pooled together in the TE estimation, and a separate weekday/weekend analysis was not performed. Table 2 reports the resulting speed, TSI and probe-vehicle-count distributions. Across the 86 segments and the study period, the number of probe vehicles contributing to a single 15 min estimate has a network-wide median of 6.4 (mean 9.6), and varies considerably between segments. Taking three probe vehicles as a threshold for low-reliability intervals, 22.3% of intervals fall below it network-wide. At the segment level, the proportion of low-sample intervals ranges from 6.2% to 74.6% (median 19.8%), with only 5 of the 86 segments exceeding 50%, predominantly minor local roads with low overall traffic volumes. Since the present analysis relies on the harmonic-mean speed rather than on traffic volumes, time intervals with few probes are expected to affect the results less than they would flow-based quantities [30]. This variability in data density is an intrinsic limitation of floating car data, but the concentration of low-sample intervals in a small subset of segments indicates that the harmonic-mean speed estimates are robust for the great majority of the dataset.

To ensure a representative sample of typical urban traffic conditions while maintaining network coherence, the road segments were selected according to the following criteria: (i) urban roads located within Catania’s city centre, (ii) homogeneous spatial distribution across the study area, (iii) segments serving primary functions in the urban network hierarchy, and (iv) exclusion of motorways and roads with speed limits exceeding 50 km/h. For each 15 min interval, the harmonic-mean speed of the available vehicle observations, calculated as the reciprocal of the mean of reciprocal individual vehicle speeds, was used to compute $TSI_{t}^{\left(i\right)}$ (Equation (2)).

The analysis was conducted separately over two daily time windows: the morning window (06:00–11:00) and the evening window (16:00–21:00).

These two windows were selected to capture distinct phases of daily urban mobility. The morning period may plausibly reflect more concentrated commuting-related movements towards central activity areas, whereas the evening period may be associated with more heterogeneous and spatially dispersed mobility patterns. Such interpretations are used only as plausible contextual explanations of the observed network differences, since the available dataset does not contain direct information on trip purpose.

After pre-processing and accounting for the required state history, the number of valid observations entering the TE calculation depends on the history length. The resulting numbers of valid observations were 798, 756, and 714 for k=1,2,3, respectively. For each road segment and analysis window, valid prediction tuples were constructed separately within each day and were subsequently pooled for probability estimation. State histories were therefore never allowed to cross the boundary between two consecutive days.

The primary analysis used the conservative discretisation b=4 and target history k=1. The same value of *b* was used in both time windows. Results for b=5,6,7 and for k=2,3 were used to evaluate sensitivity to state-space resolution and history length. For every *k*, the predicted state remains one 15 min step ahead; *k* is not interpreted as a source–target propagation delay. Statistical filtering was performed according to the surrogate-data procedure described in Section 4.3. For each history length and time window, only TE values exceeding the corresponding empirical surrogate threshold $\theta^{\left(k\right)}$ were retained in the weighted adjacency matrix $A^{\left(k\right)}\in R^{N\times N}$.

### 5.2. Results

To interpret the complex patterns uncovered by the TE analysis, we developed a suite of visualisations that capture both spatial structure and temporal evolution of the resulting networks. We investigate network connectivity through node degree and TE-based node strength, examining how these metrics vary across different history lengths and their spatial distribution. These visualisations support the identification of nodes with strong predictive influence, the characterisation of information-transfer relationships, and the assessment of structural consistency across different history lengths. Moreover, we identify the strongest information-transfer relationships that are retained across all tested history lengths within each time window.

**Figure 2:**
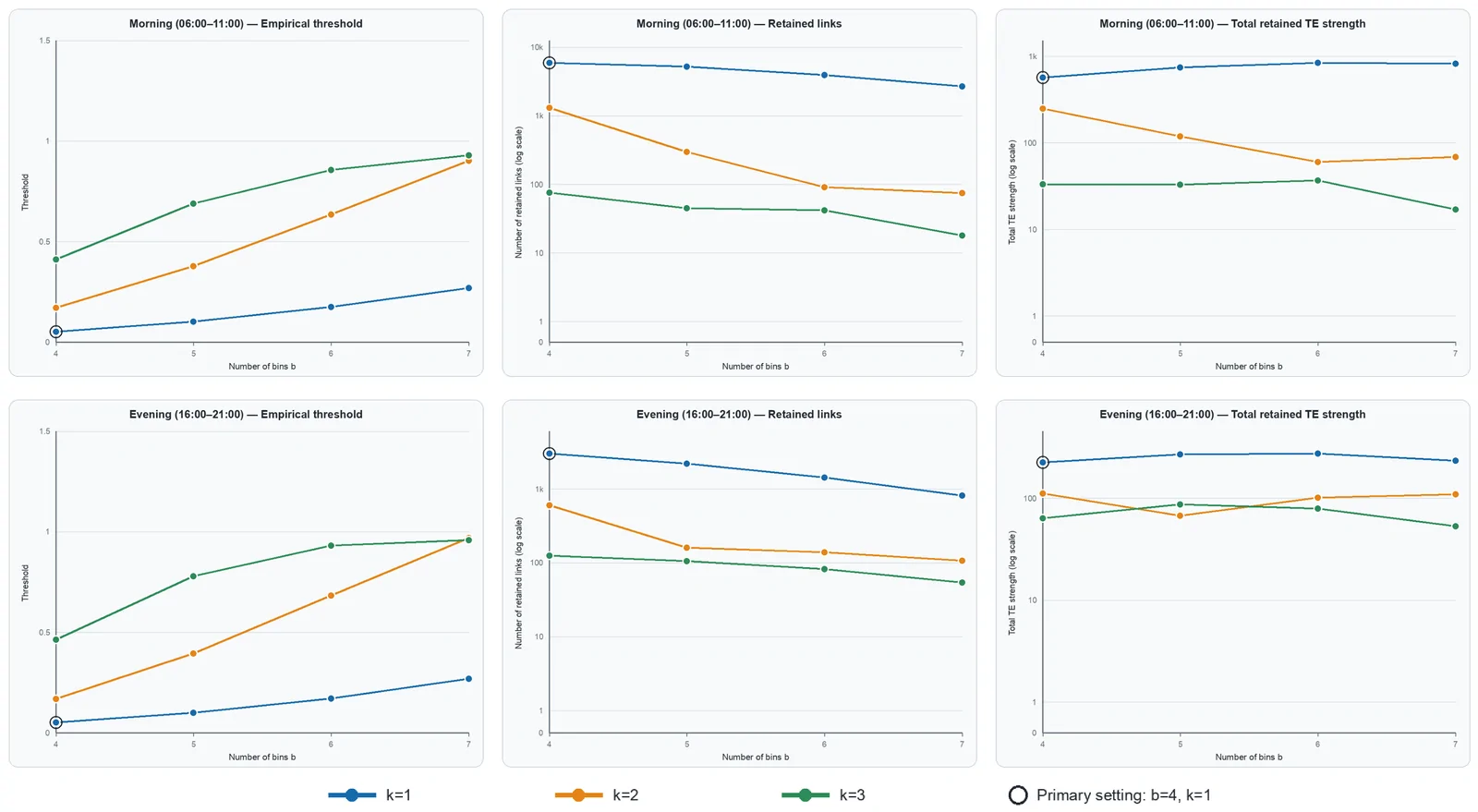
Sensitivity of the Transfer Entropy networks to the number of bins *b* and target-history length *k*. Panels report the empirical 98th-percentile surrogate threshold, the number of retained directed links, and total retained TE strength for the morning and evening windows. The source-history length is fixed at l=1. The primary specification b=4,k=1 is highlighted; k=2,3 and the remaining bin counts are treated as sensitivity analyses. Retained links exceed the pooled empirical surrogate threshold and are not interpreted as edge-wise statistically significant results.

Figure 2 summarises the sensitivity of the inferred networks to the number of bins *b* and to the target-history length *k*. For each parameter combination and time window, the figure reports the empirical 98th-percentile surrogate threshold, the number of retained directed links, and the total TE strength of the filtered network. Throughout this section, a retained link denotes a TE value exceeding the pooled empirical surrogate threshold; it should not be interpreted as an edge-wise statistically significant result.

Under the primary b=4, k=1 specification, the morning network contained 5944 retained links (81.31% of the 7310 possible directed non-self links), with a total TE strength of 565.49, whereas the evening network contained 3019 retained links (41.30%), with a total strength of 224.05. At k=2, the morning network also remained more connected and stronger than the evening network, with 1306 versus 604 retained links and total strengths of 249.39 and 110.93, respectively. At k=3, this ordering reversed: the evening network contained 125 retained links, compared with 76 in the morning, and its total strength was 63.42, compared with 33.26 in the morning. Table 3 reports the thresholds, link densities, retained TE distributions, and numbers of active source and target nodes for all six b=4 networks.

The day-block jackknife intervals in Table 3 show that the primary morning–evening contrast is not driven by any single sampled day. The 95% interval was 5621–6267 links in the morning and 2574–3464 in the evening; the corresponding intervals for total retained TE strength were 528.76–602.22 and 190.38–257.73. These network-level intervals quantify day-to-day sampling uncertainty and do not assign significance to individual links.

The sensitivity analysis shows, however, that the morning–evening contrast at k=2 depends on the discretisation parameter. The morning network is larger and stronger at b=4 and b=5, whereas the evening network becomes larger at b=6 and b=7. By contrast, the higher morning connectivity at k=1 and the higher evening connectivity at k=3 are observed throughout the explored bin range. Consequently, the k=2 comparison is interpreted as parameter-sensitive and is not used as a principal substantive conclusion. The increase in mean observed TE with *b* was accompanied by a substantial increase in surrogate TE and in the empirical filtering threshold, together with a reduction in the number of retained links. Raw mean TE was therefore not treated as an optimisation criterion. Instead, b=4 was adopted as the primary, least-sparse discretisation, while b=5,6,7 were used to assess sensitivity. Differences between the morning and evening windows may plausibly reflect changes in the temporal organisation of urban mobility, but the available data do not contain direct information on trip purpose and do not support a specific behavioural explanation.

Under the primary k=1 specification, the five nodes with the largest outgoing TE strength in the morning were IDs 13, 175, 14, 30, and 8. The corresponding highest-ranked incoming nodes were IDs 257, 212, 134, 228, and 255. In the evening, the five highest-ranked outgoing nodes were IDs 8, 2, 22, 225, and 227, whereas the highest-ranked incoming nodes were IDs 176, 76, 503, 64, and 202. These rankings identify road segments whose past states contain comparatively large amounts of predictive information about other segments, or whose future states receive comparatively large amounts of predictive information from the rest of the inferred network. They should not be interpreted as evidence of direct physical influence.

The largest retained TE value in the primary morning network was 0.2591 bits for the directed pair 13→134, whereas the largest evening value was 0.1793 bits for 13→176. Table 4 reports the five strongest source–target pairs in the primary analysis in each time window, providing their actual TE magnitudes in addition to their rankings.

The node rankings were more stable across discretisations than the exact edge topology. At k=1, comparing b=4 with b=7 yielded Spearman correlations of 0.924 and 0.800 for outgoing strength in the morning and evening windows, respectively. The corresponding edge-set Jaccard indices were substantially lower, at 0.436 and 0.214. Thus, some high-ranking source nodes recur across discretisations even though the individual links associated with those nodes are less stable.

**Figure 3:**
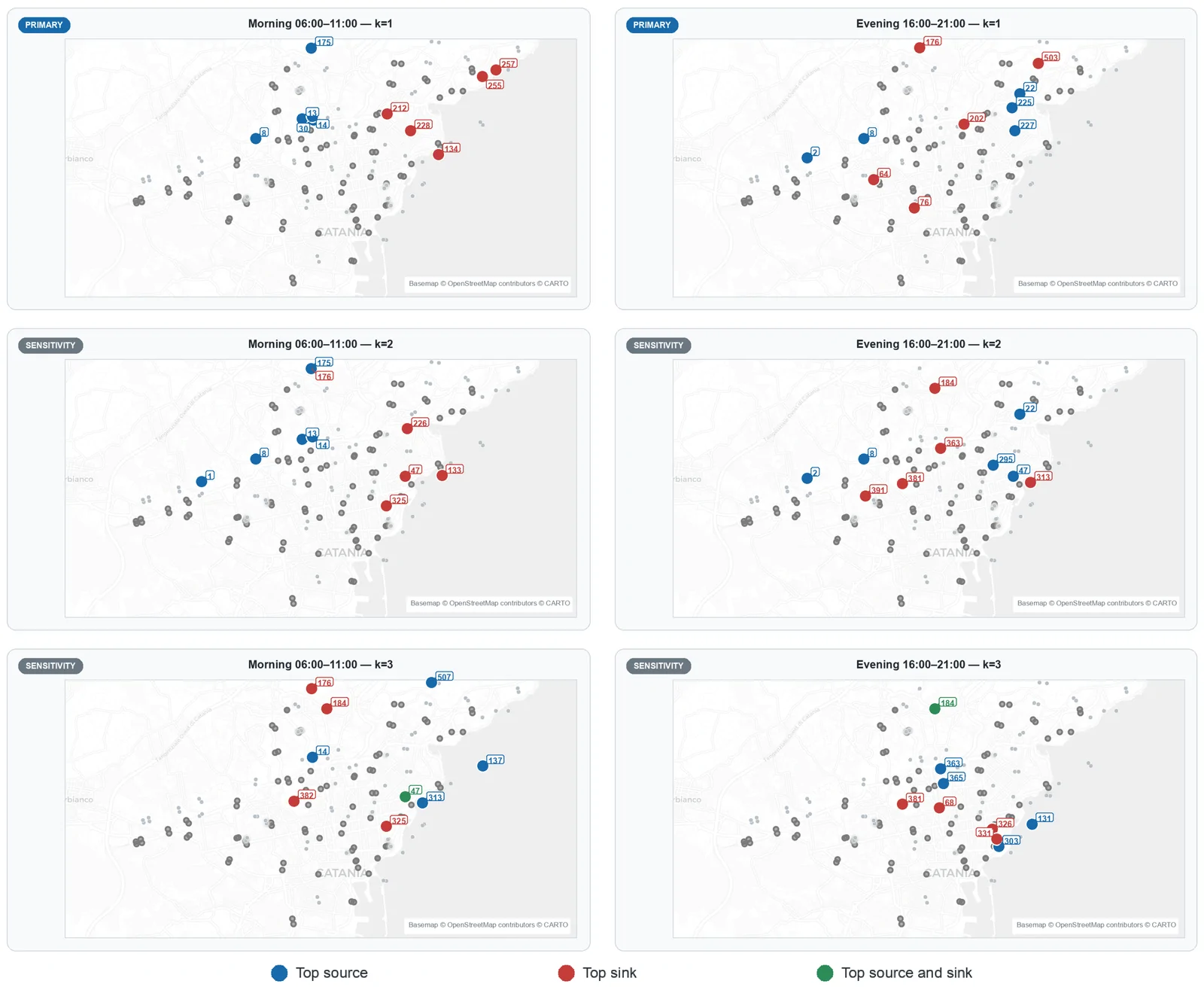
Spatial distribution of the five highest-ranked source and target nodes under the primary discretisation b=4. Blue markers identify nodes with the largest outgoing TE strength, red markers identify nodes with the largest incoming TE strength, and green markers identify nodes appearing in both rankings. Rows correspond to target-history lengths k=1,2,3, with source-history length fixed at l=1; columns compare the morning and evening windows. The k=1 panels constitute the primary analysis, whereas k=2,3 are exploratory sensitivity analyses.

Figure 3 presents the spatial distribution of the five highest-ranked source nodes and the five highest-ranked target nodes according to outgoing and incoming TE strength, respectively. All other active road segments are shown in muted grey, while the desaturated map of Catania is included solely for geographical orientation. The k=1 panels represent the primary results, whereas the k=2 and k=3 panels illustrate the sensitivity of the rankings to the amount of target history included in the estimator. Under the primary discretisation b=4, only node 14 remained among the five highest-ranked morning sources at all three values of *k*. No node remained among the five highest-ranked evening sources at all three history lengths, and no target remained in the top five across all three values of *k* in either time window. The identities of the highest-ranked nodes at longer history lengths should therefore be interpreted cautiously.

These results do not support a general claim that individual road segments maintain fixed source or target roles across all history lengths. The broader sensitivity analysis nevertheless indicates that the k=1 source rankings show partial stability across different discretisations, whereas rankings based on longer histories and the identities of the principal targets are more parameter-dependent.

**Figure 4:**
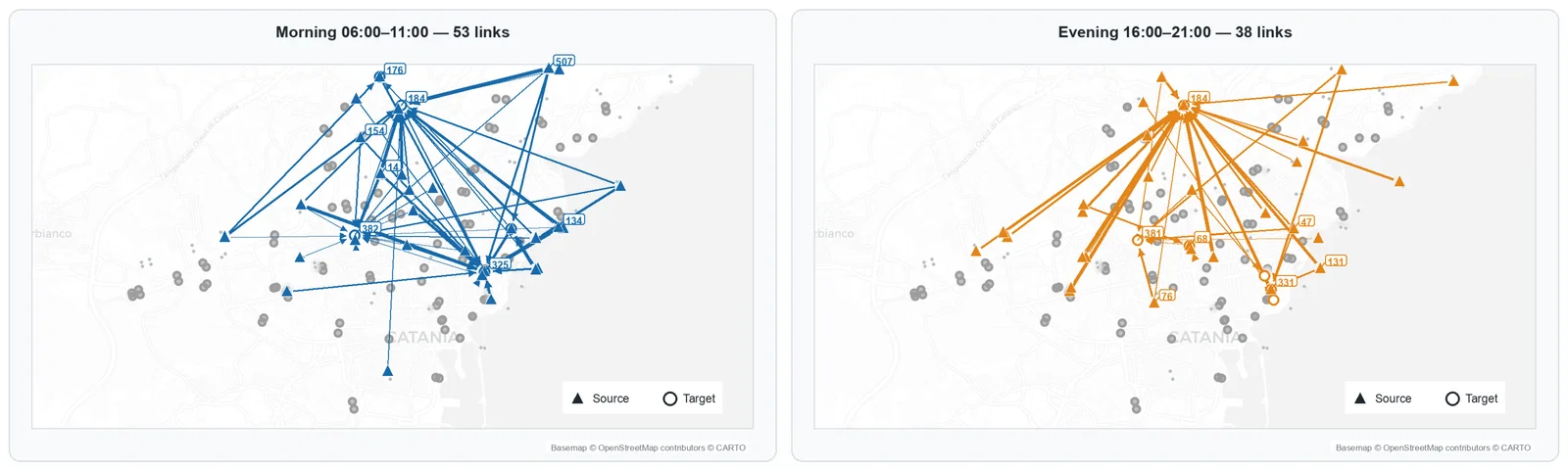
Directed information-transfer links retained at all three target-history lengths under the primary discretisation b=4 and fixed source-history length l=1. Triangular markers represent source nodes and circular markers represent target nodes. Morning and evening relationships are shown in separate panels.

Figure 4 displays the directed links retained at all three target-history lengths under the primary discretisation b=4. A total of 53 such links were identified in the morning and 38 in the evening. In the morning, most recurrent links were directed toward target nodes 382, 184, and 325. In the evening, they were directed primarily toward node 184, with smaller groups directed toward nodes 381 and 331.

Recurrence across history lengths is nevertheless strongly dependent on the number of bins. When *b* increased from 4 to 7, the number of links retained at all three values of *k* fell from 53 to 2 in the morning and from 38 to 8 in the evening. The relationships shown in Figure 4 are therefore interpreted as exploratory links recurring under the primary discretisation, rather than as parameter-independent or physically persistent connections.

### 5.3. Discussion

The sensitivity analysis changes the interpretation of the differences between the morning and evening networks. Under the primary b=4 specification, the morning network exhibits higher connectivity and greater total TE strength at k=1 and k=2, whereas the evening network exceeds the morning network only at k=3. The morning–evening ordering at k=2 changes when larger numbers of bins are used and is therefore not robust to discretisation. Accordingly, the results do not support a general conclusion that evening networks are more strongly connected for all longer history lengths.

A clearer pattern concerns the distinction between node rankings and edge-level topology. At k=1, the ranking of outgoing node strength remains comparatively stable across different bin counts, particularly in the morning, whereas the exact set of retained links and the ranking of incoming strength are less stable. This suggests that some road segments repeatedly occupy prominent informational roles even though the individual source–target relationships associated with them depend on the discretisation. Relationships recurring across k=1,2,3 should also be interpreted cautiously. Their number decreases markedly as *b* increases, demonstrating that recurrence across history lengths does not by itself establish a parameter-independent network structure.

We emphasise that TE measures informational dependencies, not vehicular flows. While flows quantify how many vehicles travel from a source segment to a target segment, TE quantifies how much the past state of the source segment improves prediction of the target segment’s future state. The TE-based links do not represent vehicle trajectories but statistical regularities in the temporal evolution of congestion states. The occurrence of retained dependencies between non-adjacent segments further indicates that TE-based connectivity does not necessarily coincide with physical road adjacency. These non-local dependencies should therefore be interpreted as predictive temporal relationships and not, by themselves, as evidence of physical congestion propagation between the corresponding road segments.

These findings suggest practical applications for traffic monitoring and traffic-management analysis:

- Rather than positioning sensors solely in the most congested areas, traffic authorities may consider nodes with high outgoing TE-based strength as candidate locations for enhanced monitoring, since their past traffic states contain predictive information about multiple other segments.
- Understanding how the informational role of individual nodes changes between time windows may support the design of time-aware monitoring and traffic-management analyses that account for daily variations in network dependencies.
- Data-driven prediction models can be improved by incorporating segment-to-segment information-transfer flow rather than relying solely on historical patterns. The directed information-transfer networks derived from Transfer Entropy can serve as input features for deep learning models, potentially improving prediction accuracy.
- This approach demonstrates that meaningful network insights can be obtained with relatively minimal data requirements and computational overhead compared to comprehensive traffic simulation models, making it accessible for cities with limited technical resources.

### 5.4. Limitations and Future Research

It is important to underline that the present analysis is based on a bivariate formulation of Transfer Entropy, which evaluates pairwise directional dependencies between source and target segments. Although this framework does not explicitly control for hidden common drivers or indirect dependencies, the inclusion of past state histories helps distinguish predictive temporal dependencies from purely instantaneous co-fluctuations. Nevertheless, retained TE links should not be interpreted as evidence of direct physical causation, since bivariate Transfer Entropy cannot fully disentangle direct dependencies from relationships induced by unobserved common factors.

Future extensions will consider conditional or multivariate TE to isolate direct from indirect dependencies and explore higher-order structures, as well as finer frameworks for parameter selection based on the Minimum Description Length principle [31].

Local heterogeneities not captured by the global quantile-based discretisation could be further explored by testing hybrid or continuous estimators [25], or by modelling congestion dynamics through self-exciting Hawkes processes. Moreover, scaling the approach to larger networks, finer temporal resolutions, and different urban morphologies will provide stronger validation. Finally, linking TE-based information flow with physical road topology and extending the framework to multimodal data—such as public transport, pedestrian, and cycling flows—represent promising steps toward a comprehensive, statistically informed understanding of urban mobility systems.

## 6. Conclusions

This study shows how Transfer Entropy can be used to characterise directional information dependencies in an urban traffic network. By applying this information-theoretic approach to floating car data from Catania, we move beyond the description of where congestion occurs and examine how the past state of one road segment provides predictive information about the future state of other segments across the network.

Several parameter-dependent patterns emerged from the analysis. Under the primary b=4, k=1 specification, the morning network contained more retained links and greater total TE strength than the evening network. Increasing the target-history length substantially reduced network density, and the relative ordering of the morning and evening networks depended on both *k* and the discretisation parameter. Some high-ranking source nodes at k=1 remained comparatively stable across different bin counts, whereas the exact edge topology, the principal target nodes, and relationships recurring across multiple history lengths were less robust. The results therefore support the identification of candidate locations with recurrent predictive importance, but not fixed causal roles or parameter-independent propagation pathways.

These findings show that the informational role of road segments varies both across history lengths and between daily time windows. Such information may support time-aware traffic monitoring by identifying locations whose traffic states exhibit recurrent predictive relationships with other parts of the network. These rankings represent informational and predictive relevance within the inferred network and should not be interpreted as evidence that intervention at a given segment would necessarily produce network-wide traffic effects.

Although the present analysis adopts a bivariate TE framework, it reveals directional information-dependence patterns that vary with the history length considered. By incorporating past state histories, the formulation captures predictive temporal dependencies beyond purely instantaneous co-fluctuations. However, this does not exclude the possible role of hidden common drivers or indirect dependencies. Nonetheless, future research will extend this approach to conditional or multivariate TE to better discriminate direct from indirect effects and to integrate higher-order information pathways.

Moreover, while the present framework treats the network as fully connected, its results reveal non-local dependencies that complement, rather than contradict, physical adjacency. Comparing these TE-based information-dependence maps with the actual road graph offers a promising direction for investigating how informational and physical connectivity are related in urban traffic networks.

## Figures and Tables

**Table 1:** Sensitivity of the Transfer Entropy results to the number of bins *b* under the k=1, l=1 specification. Mean observed TE was calculated across all directed non-self source–target pairs. The empirical threshold corresponds to the 98th percentile of the pooled surrogate distribution. Retained links are those whose TE exceeds this threshold.

Time Window	Bins *b*	Mean Observed TE	Mean Surrogate TE	Empirical Threshold	Retained Links	Total TE Strength
Morning	4	0.0849	0.0336	0.0518	5944	565.49
Morning	5	0.1252	0.0720	0.1012	5226	745.66
Morning	6	0.1804	0.1289	0.1741	3939	841.52
Morning	7	0.2487	0.2028	0.2693	2680	818.28
Evening	4	0.0515	0.0319	0.0505	3019	224.05
Evening	5	0.0873	0.0669	0.0993	2197	269.31
Evening	6	0.1359	0.1166	0.1692	1424	274.45
Evening	7	0.1983	0.1820	0.2679	811	233.22

Note: increasing *b* raises both observed and surrogate TE while reducing the number of retained links. Therefore, raw mean TE was not used as an optimisation criterion. The primary analysis adopts b=4 as the least-sparse specification, whereas b=5,6,7 are treated as sensitivity settings.

**Table 2:** Descriptive statistics of the underlying speed, Traffic Speed Index (TSI) and probe-vehicle-count data across the 86 road segments over the study period (18 September 2023–29 October 2023). Panel (a) reports statistics pooled over all valid 15 min intervals (i.e., intervals with at least one probe-vehicle observation). Panel (b) reports the distribution, across the 86 segments, of segment-level free-flow speed, mean speed, mean TSI, median probe-vehicle count, and the percentage of intervals with fewer than 3 probe vehicles. Free-flow speeds are night-time estimates and often exceed posted limits; since the TSI is capped at 1 and the discretisation is quantile-based, isolated extreme speed values do not affect the index.

(a) Network-wide pooled statistics										
Window	Valid intervals	Speed min	Speed mean	Speed max	Speed std	TSI mean	TSI std	Probe median	Probe mean	Probe max
Overall	306,885	0.34	38.06	157.62	16.86	0.740	0.219	6.4	9.62	87.0
Morning	65,568	0.92	35.53	144.95	16.62	0.694	0.229	6.25	9.77	87.0
Evening	71,806	0.50	32.11	111.93	14.41	0.645	0.202	9.67	13.13	80.67
(b) Cross-segment variability (N = 86 segments)										
Statistic	Free-flow speed (km/h)		Mean speed (km/h)		Mean TSI		Median probe count		% intervals <3 probes	
Minimum	24.4		17.3		0.55		2.0		6.2	
Median	47.9		35.0		0.74		6.3		19.8	
Maximum	91.1		83.9		0.92		26.0		74.6	

**Table 3:** Quantitative network summaries under the primary discretisation b=4 and fixed source-history length l=1. Panel (a) reports point estimates for all target-history lengths. Active sources and targets are nodes with at least one retained outgoing or incoming link, respectively. Panel (b) reports two-sided 95% leave-one-day-out block-jackknife confidence intervals for the primary k=1 analysis. TE values are expressed in bits.

(a) Point estimates									
Window	*k*	Threshold	Links (%)	Total TE	Mean TE	Median TE	Maximum TE	Sources, *n* (%)	Targets, *n* (%)
Morning	1	0.0518	5944 (81.31)	565.49	0.0951	0.0897	0.2591	86 (100.0)	86 (100.0)
Morning	2	0.1695	1306 (17.87)	249.39	0.1910	0.1863	0.2870	76 (88.4)	65 (75.6)
Morning	3	0.4110	76 (1.04)	33.26	0.4376	0.4349	0.5036	35 (40.7)	5 (5.8)
Evening	1	0.0505	3019 (41.30)	224.05	0.0742	0.0688	0.1793	82 (95.3)	83 (96.5)
Evening	2	0.1688	604 (8.26)	110.93	0.1837	0.1800	0.2471	67 (77.9)	48 (55.8)
Evening	3	0.4623	125 (1.71)	63.42	0.5074	0.4973	0.6037	49 (57.0)	8 (9.3)
(b) 95% confidence intervals for the primary k=1 analysis									
Window		Retained links	Density (%)	Total TE	Mean TE	Median TE	Maximum TE		
Morning		[5621, 6267]	[76.90, 85.73]	[528.76, 602.22]	[0.0897, 0.1005]	[0.0844, 0.0951]	[0.2006, 0.3177]		
Evening		[2574, 3464]	[35.22, 47.38]	[190.38, 257.73]	[0.0695, 0.0789]	[0.0635, 0.0741]	[0.1348, 0.2238]		

**Table 4:** Principal node and edge results under the primary b=4, k=1, l=1 specification. Source and target nodes are ranked by outgoing and incoming TE strength, respectively; the corresponding degree is the number of retained outgoing or incoming links. The lower panel reports the five directed source–target pairs with the largest retained TE values. TE values are expressed in bits.

(a) Highest-ranked source and target nodes												
	Morning						Evening					
Rank	Source	Out-strength	Out-degree	Target	In-strength	In-degree	Source	Out-strength	Out-degree	Target	In-strength	In-degree
1	13	12.368	84	257	10.828	81	8	6.502	69	176	5.949	64
2	175	11.707	83	212	9.816	80	2	6.388	70	76	5.240	61
3	14	11.560	84	134	9.813	79	22	6.045	68	503	5.229	59
4	30	11.010	84	228	9.670	79	225	5.925	68	64	4.975	59
5	8	10.882	84	255	9.291	78	227	5.804	65	202	4.582	55
(b) Directed pairs with the largest retained TE												
	Morning						Evening					
Rank			Source → target		TE		Rank		Source → target		TE	
1			13→134		0.2591		1		13→176		0.1793	
2			13→507		0.2391		2		16→176		0.1714	
3			30→134		0.2377		3		8→176		0.1612	
4			175→134		0.2298		4		16→157		0.1602	
5			13→212		0.2296		5		14→176		0.1595	

## Data Availability

The traffic data used in this study were obtained from TomTom’s Floating Car Data platform and are not publicly available. Access may be requested directly from TomTom subject to their data sharing conditions. No other datasets were generated or analysed during the current study.

## Appendix A. Road Segment Metadata

Table A1 reports the identifying, geometric and data-availability information for all 86 road segments used in the analysis.

**Table A1:** Descriptive and geometric information for all 86 road segments used in the analysis. ID: TomTom route identifier used throughout the manuscript. Street: street name. FRC: TomTom functional road class of the segment (3 = secondary road; 4 = local connecting road; 5 = local road, high importance) [29]. Lanes: number of lanes. Limit: posted speed limit (km/h). 1W: whether the segment is one-way (Y/N). Long., Lat.: WGS84 coordinates of the segment’s TomTom-provided reference point (decimal degrees). Length: TomTom-covered segment length (m). FFS: free-flow speed estimated from night-time (00:00–06:00) observations with Tukey IQR outlier removal, following the free-flow speed definition given after Equation (2) (km/h). Valid: percentage of nominal 15 min intervals within the study period (18 September 2023–29 October 2023) for which at least one probe observation was available. Probes: median number of probe vehicles per valid 15 min interval. Low-s.: percentage of valid intervals with fewer than 3 contributing probe vehicles.

ID	Street	FRC	Lanes	Limit	1W	Long.	Lat.	Length	FFS	Valid	Probes	Low-s.
1	Viale Felice Fontana	4	2	50	Y	15.04287	37.51361	261	64.0	92.6	16.6	14.1
2	Viale Felice Fontana	4	2	50	Y	15.04215	37.51437	239	64.8	96.6	16.8	10.8
7	Viale Lorenzo Bolano	4	2	50	Y	15.05676	37.51766	229	70.1	92.8	15.7	13.6
8	Viale Lorenzo Bolano	4	2	50	Y	15.05691	37.51882	312	67.8	97.5	15.5	8.4
13	Viale Andrea Doria	4	2	50	Y	15.06886	37.52337	108	61.2	98.7	23.3	6.7
14	Viale A. Usodimare	4	2	50	Y	15.07156	37.52394	196	60.9	98.7	25.3	6.2
16	Viale Andrea Doria	4	2	50	Y	15.07824	37.52566	206	63.1	98.5	21.0	7.1
21	Viale Marco Polo	4	2	50	Y	15.09816	37.52958	249	57.2	93.7	18.7	11.2
22	Viale Marco Polo	4	2	50	Y	15.09734	37.52916	247	60.1	97.7	21.5	7.7
30	Viale Andrea Doria	4	2	50	Y	15.07189	37.52311	276	57.4	97.0	26.0	10.4
31	Viale Felice Fontana	4	2	50	Y	15.03712	37.51134	276	68.5	94.8	19.9	12.3
32	Viale Felice Fontana	4	2	50	Y	15.03688	37.51235	191	58.2	98.4	22.0	8.4
33	Viale Felice Fontana	3	2	50	Y	15.02774	37.50889	499	91.1	86.5	8.5	17.1
34	Viale Felice Fontana	3	2	50	Y	15.02754	37.50951	268	81.9	92.8	11.0	16.3
35	Viale Felice Fontana	3	1	50	Y	15.02933	37.50920	367	72.1	91.6	11.0	15.4
36	Viale Felice Fontana	3	1	50	Y	15.02903	37.51001	316	67.8	93.8	8.0	15.2
45	Viale XX Settembre	4	2	50	Y	15.08765	37.51358	157	40.8	88.8	7.3	15.8
47	Corso Italia	4	2	50	Y	15.09561	37.51491	159	42.0	87.4	6.7	16.5
51	Via Palermo	4	1	50	N	15.04233	37.51039	271	51.2	89.1	4.0	24.1
52	Via Palermo	4	1	50	N	15.04297	37.51094	271	51.7	91.1	5.0	23.5
63	Viale M. Rapisardi	4	2	50	N	15.05962	37.50871	161	42.9	86.5	4.0	34.0
64	Viale M. Rapisardi	4	2	50	N	15.05940	37.50943	161	43.2	85.2	3.7	35.7
67	Viale M. Rapisardi	4	1	50	N	15.07672	37.51162	262	40.3	86.2	4.3	26.8
68	Viale M. Rapisardi	4	1	50	N	15.07643	37.51228	262	39.6	68.8	2.0	74.6
71	Corso Indipendenza	4	2	50	N	15.05431	37.50474	182	54.2	84.0	3.0	40.1
72	Corso Indipendenza	4	2	50	N	15.05463	37.50538	182	52.2	81.7	3.0	45.4
75	Viale Medaglie d’oro	5	2	50	Y	15.07034	37.50449	182	40.7	86.7	3.7	35.4
76	Viale Medaglie d’oro	5	2	50	Y	15.06993	37.50290	146	44.1	86.4	4.0	31.6
91	Via Acquicella	4	2	50	Y	15.07289	37.49179	184	58.3	76.7	2.0	58.8
92	Via Acquicella	4	2	50	Y	15.07333	37.49049	187	61.0	80.6	3.0	42.8
111	Via D. Tempio	4	2	50	Y	15.09072	37.49552	297	62.4	96.3	17.6	11.5
112	Via D. Tempio	4	2	50	Y	15.09003	37.49565	216	55.3	99.1	14.7	6.3
125	Via Sei Aprile	4	2	50	Y	15.09768	37.50557	244	49.5	98.0	15.2	9.9
126	Via Sei Aprile	4	2	50	Y	15.09756	37.50564	186	42.1	99.1	13.0	6.6
127	Via Card. Dusmet	4	2	30	Y	15.09502	37.50204	232	42.1	98.5	19.6	8.6
131	Viale Africa	4	2	50	Y	15.10050	37.50854	201	47.9	97.6	16.3	9.0
132	Viale Africa	4	2	50	Y	15.10010	37.50831	230	48.9	98.0	9.0	11.1
133	Viale Africa	4	2	50	Y	15.10519	37.51503	280	49.9	95.7	12.0	10.1
134	Viale Africa	4	2	50	Y	15.10429	37.51517	229	45.4	97.6	10.2	9.8
135	Viale R. di Lauria	4	2	30	N	15.10745	37.51788	196	38.6	94.6	11.4	10.1
137	Viale R. di Lauria	4	2	30	N	15.11575	37.52191	182	38.4	94.3	10.0	10.3
138	Viale R. di Lauria	4	2	30	N	15.11509	37.52262	182	42.7	96.5	9.5	10.8
153	Via Santa Sofia	4	2	30	Y	15.06870	37.53010	182	50.1	81.1	4.5	29.3
154	Via Santa Sofia	4	2	30	Y	15.06787	37.52981	228	48.2	74.8	3.0	38.0
157	Via Santa Sofia	4	2	50	Y	15.06793	37.53551	152	48.5	84.3	5.6	25.4
158	Via Santa Sofia	4	2	50	Y	15.06705	37.53611	132	48.4	83.0	4.0	29.9
175	Via del Fasano	4	2	50	Y	15.07127	37.53965	200	47.3	90.5	9.0	14.9
176	Via del Fasano	4	2	50	Y	15.07137	37.53971	216	43.1	88.7	6.0	20.0
183	Via Passo Gravina	4	2	50	Y	15.07479	37.53447	202	52.8	91.4	10.2	13.9
184	Via Passo Gravina	4	2	50	Y	15.07535	37.53509	198	44.0	88.4	5.3	20.4
187	Via Passo Gravina	5	2	50	Y	15.08114	37.52159	215	32.3	87.4	7.0	17.7
202	Via Etnea	4	1	40	Y	15.08285	37.52219	244	41.9	88.5	5.8	19.7
211	Via V. Giuffrida	4	3	50	Y	15.09081	37.52432	167	40.6	95.8	24.0	10.7
212	Via V. Giuffrida	4	3	50	Y	15.09095	37.52454	149	44.3	95.2	20.4	9.5
225	Viale Vittorio Veneto	4	2	50	N	15.09534	37.52588	217	47.3	87.2	6.6	22.8
226	Viale Vittorio Veneto	4	2	50	N	15.09620	37.52579	217	49.1	85.9	4.8	26.6
227	Viale Vittorio Veneto	4	2	50	N	15.09605	37.52061	181	44.6	92.6	10.0	16.0
228	Viale Vittorio Veneto	4	1	50	N	15.09703	37.52061	181	47.1	92.3	7.3	16.8
235	Via Androne	5	1	50	Y	15.08085	37.51033	245	38.9	85.3	5.0	25.1
247	Viale Giuseppe Laino	5	2	50	Y	15.11133	37.53753	305	52.7	80.7	5.0	27.3
248	Viale Giuseppe Laino	5	2	50	Y	15.11199	37.53693	213	52.6	77.9	4.0	31.3
251	Via Acicastello	5	2	50	Y	15.12240	37.53464	174	53.1	84.6	6.0	19.9
253	Via Nazionale	4	1	50	N	15.12502	37.53896	335	59.5	88.7	8.8	14.7
254	Via Nazionale	4	1	50	N	15.12484	37.53984	335	61.4	93.3	8.0	14.1
255	Via Acicastello	4	2	50	Y	15.11562	37.53308	293	55.0	94.3	16.8	11.9
257	Via Antonino Caruso	4	2	50	Y	15.11914	37.53464	133	48.9	91.6	11.0	12.4
295	Via G. D’Annunzio	5	2	50	Y	15.09042	37.51742	205	34.0	85.3	4.9	26.5
302	Via V. Emanuele II	5	1	50	Y	15.08046	37.50192	221	34.3	77.6	2.2	60.1
303	Via V. Emanuele II	5	1	30	Y	15.09190	37.50344	208	32.2	91.5	5.0	24.8
311	Via Umberto I	5	1	30	Y	15.08711	37.51141	249	28.6	84.3	3.7	38.9
313	Via Umberto I	5	1	30	Y	15.10015	37.51344	163	27.7	76.3	3.0	43.7
325	Corso Sicilia	4	2	50	Y	15.09073	37.50802	249	28.0	87.1	4.0	33.2
326	Corso Sicilia	4	2	50	Y	15.09024	37.50737	253	31.2	85.6	3.7	37.2
331	Via A. di Sangiuliano	5	1	50	N	15.09140	37.50514	277	29.5	83.9	3.0	44.1
332	Via A. di Sangiuliano	5	1	50	N	15.09121	37.50502	277	24.4	82.3	2.8	51.5
361	Viale A. Fleming	5	2	50	Y	15.07546	37.52367	260	44.4	80.7	6.0	22.6
363	Viale A. Fleming	5	2	50	Y	15.07684	37.52126	179	45.2	80.7	5.8	23.4
365	Via Milo	5	2	50	Y	15.07756	37.51790	326	40.6	82.0	5.0	27.1
381	Via S. M. Castaldi	5	2	50	Y	15.06687	37.51313	162	54.1	85.2	3.8	34.2
382	Via S. M. Castaldi	5	2	50	Y	15.06679	37.51386	165	45.3	78.7	3.0	48.0
391	Via Armando Diaz	5	1	50	N	15.05734	37.51037	167	43.3	74.3	2.7	51.4
392	Via Armando Diaz	5	1	50	N	15.05668	37.51031	167	42.0	75.7	3.0	49.7
503	Via Nizzeti	5	1	50	N	15.10214	37.53613	250	47.8	83.0	5.9	22.6
504	Via Nizzeti	5	1	50	N	15.10106	37.53644	250	49.0	87.3	7.0	19.6
507	Via Venti Settembre	5	1	40	N	15.10250	37.54109	144	36.9	77.0	4.0	32.1
512	Via Sgroppillo	5	1	50	N	15.10442	37.54089	259	46.5	83.0	5.0	25.5
